# Optimization of pre‐analytical and analytical steps for DNA and RNA analysis of fresh cytology samples

**DOI:** 10.1002/cam4.4728

**Published:** 2022-04-10

**Authors:** Ana Dolinar, Gašper Grubelnik, Irena Srebotnik‐Kirbiš, Margareta Strojan Fležar, Margareta Žlajpah

**Affiliations:** ^1^ Department of molecular genetics, Institute of Pathology, Faculty of Medicine University of Ljubljana Ljubljana Slovenia; ^2^ Department of cytopathology, Institute of Pathology, Faculty of Medicine University of Ljubljana Ljubljana Slovenia

**Keywords:** automated extraction, cytological samples, DNA, extraction methods, extraction with TRI reagent, RNA, spin‐column extraction

## Abstract

**Background:**

Different cytology preparations can be used for molecular diagnostics, however the influence of pre‐analytical and analytical steps on the results are not yet well defined. We aimed to determine optimal steps for efficient extraction of DNA and RNA from fresh cells for molecular diagnostics.

**Methods:**

MCF7 and FaDu human cell lines, were used as a model to determine fresh cells storage conditions (temperature: 25°C, 4°C, −20°C, −80°C; duration: 0 h, 4 h, 12 h, 24 h, 48 h) and optimal nucleic acids extraction method. Besides, the minimal number of total cells and minimal percentage of mutated cells needed for successful extraction of nucleic acids and subsequent determination of present mutation were evaluated.

**Results:**

Extraction of nucleic acids using spin columns yielded the highest quantity and quality of nucleic acids. Isolation of nucleic acids was feasible in all storage conditions, however higher temperature and longer duration of fresh cells storage were associated with lower quality of isolated nucleic acids and similar quantification cycle of housekeeping genes. Successful molecular testing was feasible with least 10^4^ cells, while specific mutation was detected in as low as 5% of mutated cells.

**Conclusions:**

Our cell line model, mimicking fresh cytology samples, showed that quantity of extracted either DNA or RNA declined with higher temperatures and longer duration of storage but regardless of the storage conditions, we successfully detected both housekeeping genes and mutated gene using qPCR.

## Graphical abstract

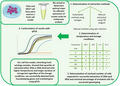

## 1 INTRODUCTION

Molecular testing is becoming more and more important in cytopathology as novel target therapies are emerging.^1^, ^2^ Reliable results are critical for the therapy selection; however, the quality of any molecular test is strongly dependent on pre‐analytical and analytical steps. While pre‐analytical steps for molecular tests on formalin‐fixed paraffin‐embedded (FFPE) tissue samples are relatively standardized, the variety of cytological sample preparation techniques poses a challenge for standardized molecular testing. Numerous studies already compared different extraction methods of nucleic acids from various cytological samples,^3^, ^4^, ^5^ however there is limited data for extraction of nucleic acids from fresh unfixed cell samples.^6^, ^7^, ^8^

Different cytological preparations have already been described for use in molecular testing: smears, cell blocks, cytospins, and liquid‐based cytology (LBC) samples, each with its advantages and disadvantages for molecular testing.^9^, ^10^ Smears are readily prepared and enable rapid on‐site evaluation (ROSE) of the sample. Various stained smears of diverse samples were already used for successful analysis of mutations, gene fusions, and miRNA expression levels.^11^, ^12^ Smear fixation process is usually alcohol‐based, leading to lower degradation of nucleic acids than formaldehyde‐based fixation^13^ beside that laser capture or manual microdissection can be applied for tumor cell enrichment.^12^ Cytospins are used to concentrate cells from cell suspensions to a designated area where further processing is usually similar to smears. Several types of samples are suitable for cytospin preparations and subsequent molecular analyses.^14^, ^15^, ^16^ Liquid‐based cytology (LBC) samples are immediately fixed cells that are further processed in the laboratory yielding monolayer slide.^17^ Successful molecular analyses were performed using LBC samples where cells are either scrapped off from slides or used directly as fixed liquid samples.^18^ Cell blocks are formalin‐fixed paraffin‐embedded (FFPE) cytology samples, thus resembling histological samples regarding the preparation technique and can be used as standard FFPE tissue samples. Several molecular tests were successfully performed using cell blocks as a source,^19^, ^20^, ^21^ however, shorter amplicons should be considered to avoid test failures due to formalin‐induced fragmentation of nucleic acids. Most frequently reported limitation of cell blocks is its low cellularity.^22^

Beside different cytology preparations, fresh cytology samples can also be used molecular testing. Their biggest advantage is that the extracted nucleic acids are not affected by fixation and other steps in processing procedure and do not require any scraping or paraffin removal.^9^

In our laboratory, highly effective standardized processing of cytology samples for different ancillary diagnostic methods was developed. After preparation of direct smears for ROSE and cytomorphological evaluation from fine‐needle aspiration biopsies (free‐hand and image‐guided) or effusions, the sample leftovers are suspended in a buffer‐based cell medium (BBCM) with bovine serum albumin (BSA) and ethylene diamine tetraacetic acid (EDTA) and stored at room temperature.^23^

These cell suspensions, prepared from different cytology samples and stored at RT, can be used in a timeframe of up to 72 h for the preparation of additional cytospins for immunocytochemistry,^24^, ^25^, ^26^ FISH^27^ or special staining’s as well as for flow cytometry immunophenotyping^28^, ^29^ or even for the preparation of cell blocks.^30^ Moreover the suitability of each cell suspension for further processing is evaluated using ROSE on test cytospin.

For our daily practice it would be very convenient to use fresh cytology samples suspended in buffer‐based media also for diagnostic, prognostic, or predictive markers testing using NGS. According to reports from the literature extraction of nucleic acids from fresh cells requires immediate processing.^31^ However, the timeframe between sample acquisition and pathologist's diagnosis with indication for molecular genetic testing is usually between 24 and 48 h. Therefore, we decided to evaluate the influence of temperature and duration of storage of fresh cytology samples in buffer‐based cell medium on the quality and quantity of nucleic acids. Moreover, to prevent unsuccessful molecular testing of cytology samples we wanted to determine minimal sufficient amount of cells and minimal percentage of tumor cells where detection of a mutation was still possible.

## 2 METHODS

### 2.1 Study design

The study was performed in three separate experiments using cells from cell lines as model mimicking real cytology samples. In first experiment, the efficiency of three DNA and RNA extraction methods (automated extraction using cartages, manual extraction based on TRI reagent, and manual extraction using spin columns) was evaluated using aliquots of 1 × 10^6^ cells from two cell lines (MCF7 and FaDu). Extractions were performed in 3 consecutive days, from separate cell harvests.

In second experiment, the influence of temperature and duration of fresh cells storage on the quality and quantity of isolated RNA was determined. Tested temperature points for stored cells were room temperature (*T* = 25°C), for 0, 4, 12, 24, and 48 h, refrigerator temperature (*T* = 4°C) for 4, 12, 24, and 48 h and at two freezer temperatures (*T* = −20°C and *T* = −80°C) for 24 h and 48 h. Then, cell's nucleic acids were extracted. The experiment was conducted with both cell lines' aliquots containing 1 × 10^6^ cells, separately. Isolated RNA was also used for quantitative real‐time polymerase chain reaction (qRT‐PCR) to determine the effects of storage time and temperature on expression levels of two housekeeping genes and one gene of interest.

In third experiment, the minimal number of cells required for successful extraction with enough quantity of DNA and RNA for molecular analysis was determined. Besides that, the minimal percentage of mutated cells for successful genotyping was determined by using MCF7 cells as a tracer cells expressing *SMAD4* gene with background cell population of FaDu cells with absent *SMAD4* expression. Percentage of tracer cells varied between 0% and 100% in a total population of 1 × 10^5^ cells. All experiments were performed in three technical replicates.

### 2.2 Cell lines

Pharyngeal squamous cell carcinoma cell line FaDu (ATCC® HTB‐43™) was cultured in RPMI 1460 medium (Life Technologies), while human breast cancer cell line MCF‐7 (ATCC® HTB‐22™) was cultured in advanced minimum essential medium (AMEM; Life Technologies). Both media were supplemented with 5% fetal bovine serum (Thermo Fisher Scientific), 10 mM/L L‐glutamine (Thermo Fisher Scientific), 100 U/mL penicillin (Grünenthal, Germany), and 50 mg/ml gentamicin (Krka). Cell lines were incubated at 37°C in a 5% CO_2_ humidified atmosphere and harvested when they reached 80% confluence.

### 2.3 Cell line preparation and determination of cell quantity

Harvested cells were centrifuged for 5 min at 1200 *g*, the pellet was washed twice using phosphate‐buffered saline (PBS; EMD Biosciences Inc.) and processed or stored according to the protocol. The density of each cell suspension in buffer‐based cell medium was assessed using Neubauer hemocytometer.

### 2.4 DNA/RNA extraction methods

#### 2.4.1 Automated method using cartages

Automatic extraction of DNA and RNA from cell samples was performed using Maxwell® RSC Instrument. Two aliquots of triplicates for each cell line were used, one for DNA extraction and one for RNA extraction.

DNA was extracted using Maxwell® RSC Cell DNA kit. Cell pellet was resuspended in 100 μl of PBS (EMD Biosciences Inc.) and the suspension was then processed according to the manufacturer's instructions.

Maxwell® RSC simplyRNA Cells kit was used for RNA extraction. Cell pellet was resuspended in 200 μl of chilled 1‐Thioglycerol/Homogenization Solution and homogenized using TissueLyser (2 min, 50 Hz) (Qiagen GmbH). Lysate was then processed according to the manufacturer's instructions. Maxwell instrument and reagents were from Promega Corporation, USA.

#### 2.4.2 Manual method based on TRI reagent

The cell pellet from one aliquot of each cell line was resuspended in 500 μl of QIAzol Lysis reagent (Qiagen GmbH, Germany) and homogenized using the TissueLyser (2 min, 50 Hz) (Qiagen GmbH). For dissociation of nucleoprotein complexes 200 μl of chloroform was added. Mixture was then vortexed vigorously for 15 s and centrifuged at 16,168 *g* for 45 min at 4°C. Centrifugation separates the mixture into three phases: a red organic phase (containing protein), an interphase (containing DNA), and a colorless upper aqueous phase (containing RNA).

For RNA extraction, aqueous phase was transferred to a fresh tube. Equal amount of 2‐propanol was added to the mixture and centrifuged at 16,168 *g* for 45 min at 4°C. After centrifugation, the supernatant was removed and the pellet was dissolved in 1 ml of 75% (v/v) ethanol. The mixture was centrifuged at 16,168 *g* for 10 min at 4°C. After centrifugation, the supernatant was removed and the RNA‐containing pellet was air dried. The RNA‐containing pellet was dissolved in 50 μl of RNAse‐free H_2_O.

For DNA extraction, interphase was transferred to a fresh tube. After adding 0.15 ml of absolute ethanol, the mixture was mixed by inversion, allowed to stand for 3 min and centrifuged at 1964 *g* for 5 min at 4°C. DNA‐containing pellet was washed twice by removing the supernatant, adding 500 μl of 0.1 M trisodium citrate in 10% ethanol, vortexing on thermomixer at 600 rpm for 15 s at 18°C and centrifuged at 1964 *g* for 5 min at 4°C. Afterwards the pellet was resuspended in 1 ml of 75% (v/v) ethanol and the mixture was put on the thermomixer for 20 min at 18°C, where it was vortexed every 5 min for 15 s at 600 rpm. Then the mixture was centrifuged at 1964 *g* for 5 min at 4°C. After centrifugation, the supernatant was removed and the DNA‐containing pellet was air dried. Dry pellet was dissolved in 300 μl 8 mM NaOH and centrifuged at 16,168 *g* for 10 min at 4°C. The supernatant containing the DNA was transferred to a fresh tube.

#### 2.4.3 Manual method using spin columns

DNA and RNA were extracted from one aliquot of cells using AllPrep DNA/RNA/miRNA Universal kit (Qiagen). Cells were disrupted in 350 μl of Buffer RLT Plus using TissueLyser (2 min, 50 Hz) (Qiagen) and all following steps were performed according to the manufacturer's instructions.

### 2.5 Quantification and purity of RNA and DNA extracts

Quantity of RNA and DNA was assessed fluorometrically on Qubit 3.0 using RNA HS assay and dsDNA HS assay (all Thermo Fisher Scientific Inc.). Samples were diluted accordingly if needed. The quality of isolated nucleic acids was assessed spectrophotometrically on NanoDrop‐1000 (Thermo Fisher Scientific Inc.) by *A*_260_/*A*_280_ and *A*_260_/*A*_230_ absorbance ratios. Nucleic acids absorb at 260 nm, proteins at 280 nm, and organic compounds (phenol, guanidine) at 230 nm. *A*_260_/*A*_280_ absorbance ratio value 1.8 is generally accepted as “pure” for DNA and 2.0 for RNA. *A*_260_/*A*_230_ absorbance ratios between 1.8 and 2.2 is generally accepted as “pure” for both DNA and RNA.

### 2.6 Design of primers for distinguishing MCF7 and FaDu cell lines

According to the ATCC guides MCF7 and FaDu cell lines possess two distinct mutations that result in lack of protein. MCF7 cell line has a mutation in the *CDKN2a* gene (ENST00000304494.9; c.1_471del471 p.?) and the FaDu cell line in *SMAD4* gene (ENST00000342988.7; c.1_1659del1659 p.?). Using Ensembl (release 98, GRCh38.p13 assembly), we have designed our primers so that we were able to detect a specific cell line in a mixture of cell lines using qRT‐PCR, respectively.

### 2.7 Reverse transcription and quantitative polymerase chain reaction

Reverse transcription was performed using LunaScript RT SuperMix Kit (New England Biolabs Inc.) according to the manufacturer's instructions. Each reaction contained 10 ng of extracted total RNA. Following cDNA synthesis, qRT‐PCR was performed using Sybr Green technology. Each reaction contained 3 ng of cDNA along with forward and reverse primers (final concentration 0.1 μM) (Table 1) and PowerUp Sybr Green Master Mix (Thermo Fisher Scientific Inc). Thermal conditions were as follows: hold for 2 min at 50°C, second hold for 2 min at 95°C followed by 45 cycles of 15 s at 95°C, 60 s at 60°C. Afterwards melting curves were acquired on the SYBR channel between 60 and 95°C using a ramping rate of 0.7°C/60 s to determine specificity of qRT‐PCR products.

**TABLE 1:** Primers for qRT‐PCR

Gene	Forward primer	Reverse primer
*GAPDH*	5`‐GAAGGTGAAGGTCGGAGTCAAC‐3`	5`‐CTTTACCAGAGTTAAAAGCAGC‐3`
*ACTB*	5`‐AACCGCGAGAAGATGACCCA‐3`	5`‐GATAGCACAGCCTGGATAGCAAC‐3`
*CDKN2a*	5`‐GCTGCCCAACGCACCGAATAG‐3`	5`‐CGCTGCCCATCATCATGAC‐3`
*SMAD4*	5`‐CCAATGTCCACAGGACAGAG‐3`	5`‐CACCTTTACATTCCAACTGC‐3`

Expression of *HPRT* was determined by qPCR using TaqMan technology. qPCR reactions were performed using TaqMan technology with FastStart Essential DNA Probe Master (Roche Diagnostics, Switzerland). Each qPCR reaction contained 1 ng cDNA, 2x FastStart Essential DNA Probe Master (Roche Diagnostics, Switzerland) and 20x TaqMan *HPRT* reference gene expression assay. The thermocycling conditions for gene expression were 10 min at 95°C and 40 cycles of 15 s at 95°C and 1 min at 60°C. The signal was collected at the endpoint of every cycle.

### 2.8 Sequencing for tested genes sequence and mutation conformation

SeqStudio Genetic Analyzer was used for determining specific mutation in each cell line by sequencing (Applied Biosystems, Thermo Fisher Scientific Inc.). qRT‐PCR products obtained by qRT‐PCR as described above, were purified with ExoSAP‐IT™ PCR Product Cleanup Reagent (Applied Biosystems, Thermo Fisher Scientific Inc.) and used for a sequencing reaction, according to manufacturer's instructions. The sequencing reaction was performed using Big‐Dye terminator chemistry version 1.3 (Applied Biosystems, Thermo Fisher Scientific Inc.) with sequencing primer (5`‐CTGAGTATTGGTGTTCCATTGCTTA‐3`). Electropherograms were analyzed using the Sequence Scanner Software 2, Version: 2.0 (Applied Biosystems, Thermo Fisher Scientific Inc.).

### 2.9 Calculations and statistical analysis

All calculations (averages, standard deviations [SD]) and graphical representation of data were done in Excel (Microsoft Corporation) and RStudio IDE (RStudio). Statistical analyses were performed in SPSS 24.0 (IBM). We used Spearman's rank correlation coefficient to calculate the correlations between time, temperature, and gene expression. All statistical analyses were performed using cut‐off value (*p*) 0.05.

## 3 RESULTS

### 3.1 Efficiency of extraction methods

**FIGURE 1:**
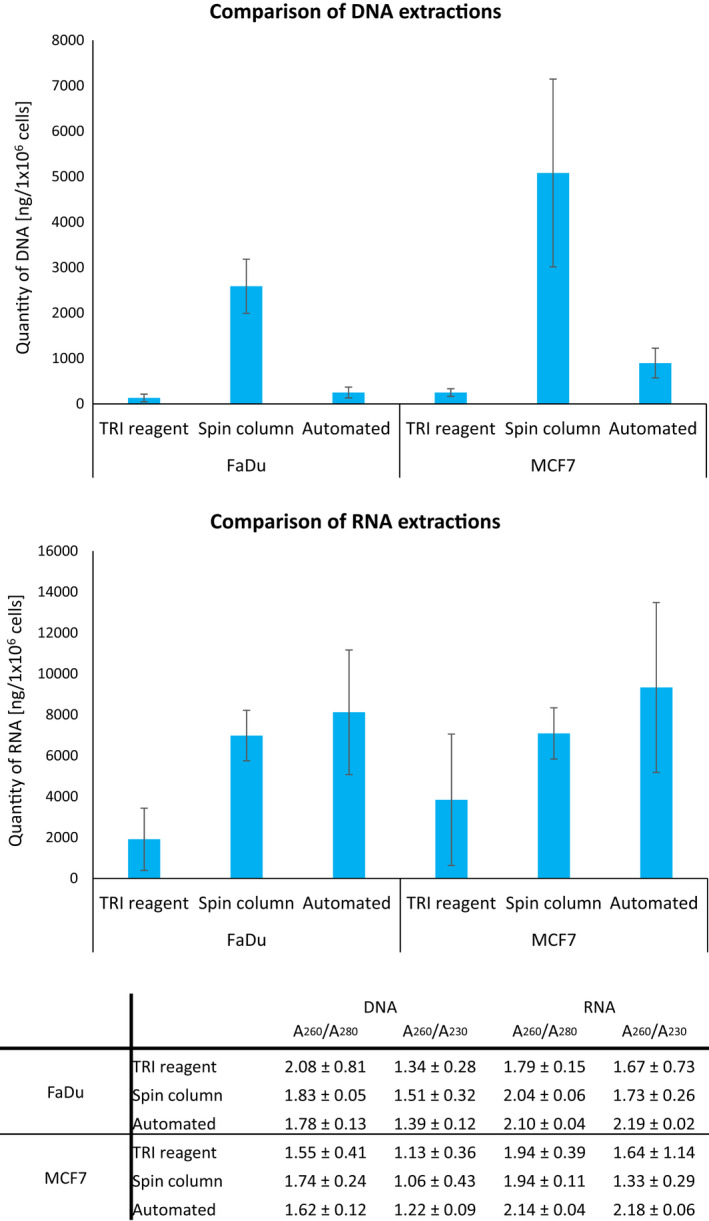
Comparison of DNA and RNA extractions using different methods in two cell lines, FaDu, and MCF7. (A) DNA and RNA quantities from extractions that were performed on three separate cell harvests. Quantities are normalized to 1 × 10^6^ cells per extraction and shown as an average 土 standard deviation. (B) Quality of obtained DNA and RNA samples. Shown are *A*_260_/*A*_280_ and *A*_260_/*A*_230_ ratios as an average 土 standard deviation. *N* = 9 for each data point

The highest DNA quantity was obtained with manual method using spin columns, while highest RNA quantity was obtained with automated method using cartages (Figure 1). DNA extraction with manual method based on TRI reagent and with automated method using cartages resulted in comparable quantities. RNA extraction with manual method using spin columns resulted in higher RNA quantity than extraction with automated method.

Minimal differences in the quality of isolated DNA and RNA from both cell lines were observed when comparing all three extraction methods, as seen from *A*_260_/*A*_280_ to *A*_260_/*A*_230_ ratios^32^ in Figure 1B.

DNA and RNA quantities were the most consistent for both cell lines when manual method using spin columns was used for extraction and was thus selected for further experiments.

### 3.2 The influence of sample storage conditions on the quality of DNA and RNA

**FIGURE 2:**
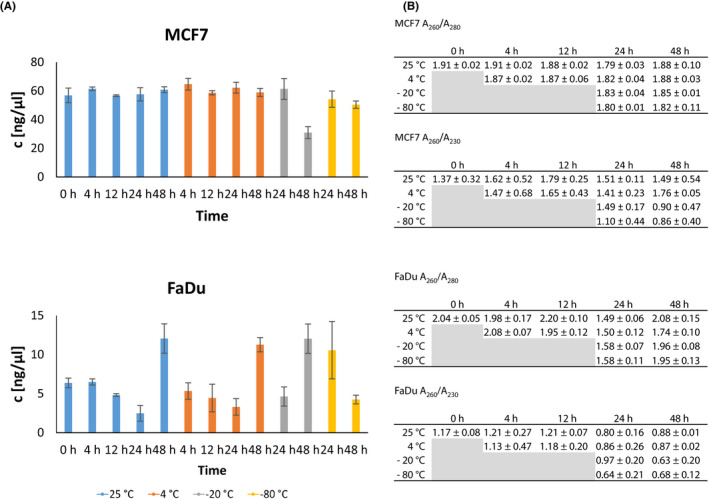
The influence of sample storage conditions on the quality of DNA. (A) DNA concentrations isolated from MCF7 and FaDu cell lines after storage, shown as an average concentration (ng/μl) 土 standard deviation. (B) Purity of isolated DNA determined by *A*_260_/*A*_280_ and *A*_260_/*A*_230_ ratios as an average 土 standard deviation. *N* = 3 for each data point

The effects of sample storage conditions were cell line dependent and more pronounced on RNA than on DNA level. Higher variability in DNA quantity was observed for extractions from FaDu cell line where the quantity dropped linearly at the same storage temperature in the first 24 h. Only minimal difference was observed for extractions from MCF7 cell line, irrespective of storage temperature or time (Figure 2). DNA from MCF7 cell line also showed higher and less variable purity compared to DNA from FaDu cell line, where slight protein contamination is possible (Figure 2B). Contamination with organic compounds was present in both cell lines as seen from A260/A230 ratio.

**FIGURE 3:**
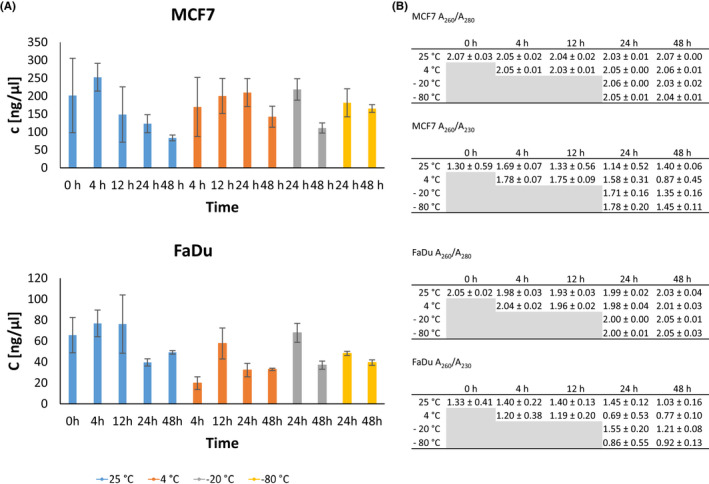
The influence of sample storage conditions on the quality of RNA. (A) RNA concentrations isolated from MCF7 and FaDu cells after storage, shown as an average concentration (ng/μl) 土 standard deviation. (B) Purity of isolated RNA determined by *A*_260_/*A*_280_ and *A*_260_/*A*_230_ ratios as an average 土 standard deviation. *N* = 3 for each data point

Compared to DNA, RNA showed higher variability in its concentrations for both cell lines (Figure 3). Room temperature affected RNA quantities the most, while lowest variation of extracted RNA quantities was observed within the samples, stored at −80°C. Purity of extracted RNA was good, irrespective of storage temperature or time, however contamination with organic compounds was present in both cell lines (Figure 3B).

**FIGURE 4:**
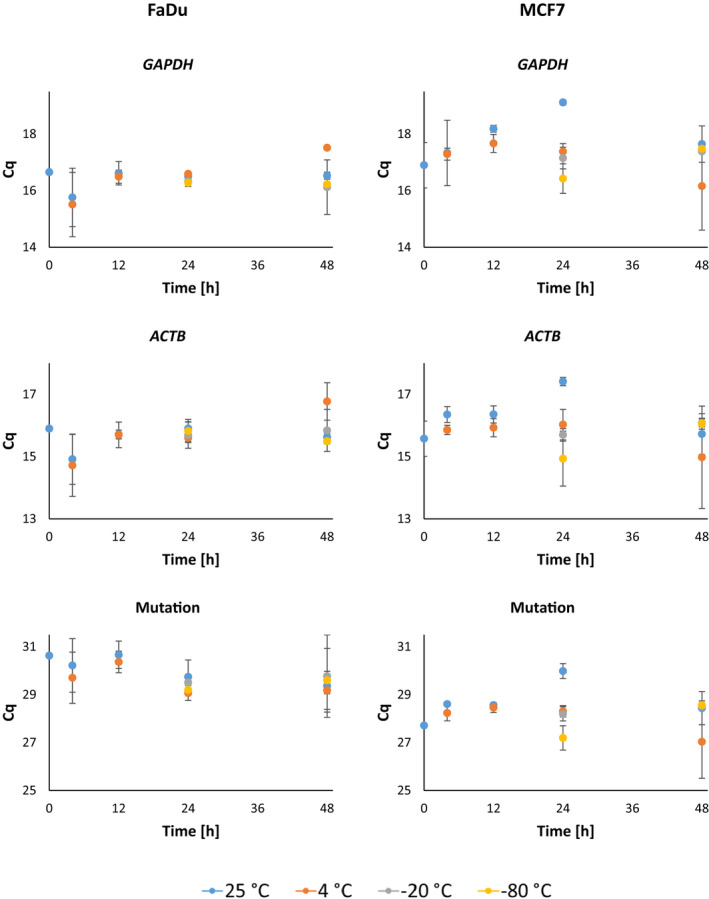
Changes in gene expression levels as an effect of different sample storage conditions. Cq, quantification cycle

**FIGURE 5:**
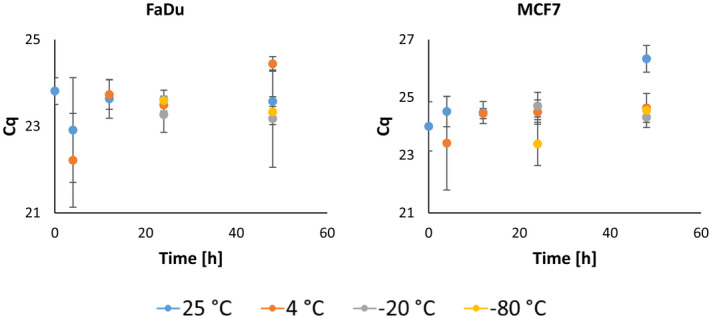
Changes in gene expression level of *HPRT* as an effect of different sample storage conditions. Cq, quantification cycle

The expression levels of housekeeping genes *GAPDH* and *ACTB* were most stable at the storage temperature −80°C, regardless of storage time (Figure 4). Expression of low‐expression gene *HPRT* did not vary over the tested period of time regardless of the storage temperature (Figure 5). Expression levels of housekeeping genes at other storage temperatures changed over a specified time range of storage; however, the variation never exceeded more than two quantification cycles. The *GAPDH* and mutation gene (*SMAD4* for MCF7 cell line and *CDKN2a* for FaDu cell line) expression levels were significantly correlated (*ρ* = 0.231, 0.042 and 0.226, 0.047) to storage temperature. The expression level of *ACTB* was statistically significantly correlated to the time of storage (*ρ* = 0.311, 0.006), while there was no correlation between the expression of HPRT and time or temperature.

### 3.3 Minimal number of cells required for successful extraction of DNA and RNA

Extraction of nucleic acids from all triplicates was successful with 10^3^ or more cells in the sample. If the number of cells was lower, the extraction of nucleic acids might be partially successful or unsuccessful. Obtained quantities increased with the number of cells in the sample, and quantities from 10^3^ cells were on the limit of the detection (Table 2).

**TABLE 2:** RNA and DNA quantities from different numbers of cells from MCF‐7 and FaDu cell lines, shown as an average (ng/μl) 土 SD. *N* = 3, unless specified otherwise

Number of cells	RNA (ng/μl)		DNA (ng/μl)	
	MCF7	FaDu	MCF7	FaDu
10^5^	204.20 土 11.82	178.53 土 16.57	49.96 土 12.36	56.56 土 6.85
10^4^	25.07 土 2.00	23.02 土 3.82	4.90 土 0.49	4.64 土 0.52
10^3^	3.24 土 0.20	2.57 土 0.07	0.35 土 0.05	0.30 土 0.07
10^2^	1.33 土 0.00 (*N* = 2)	1.40 (*N* = 1)	/ (*N* = 0)	0.04 土 0.01 (*N* = 2)

### 3.4 Minimal percentage of mutated cells for successful genotyping

Successful genotyping of testing gene *SMAD4* was ensured with as low as 5% of tracer cells in the sample (Table 3) and the expression levels of testing gene were correlated with the percentage of tracer cells in the sample.

**TABLE 3:** Average quantification cycles and standard deviations of qRT‐PCR products of *SMAD4* transcript from 5% to 100% of tracer cells from MCF7 cell line. Zero percent of cells from MCF7 cell line represents 100% of background cells from FaDu cell line

MFC7 (%)	Average quantitation cycle (Cq)	Standard deviation
0	Not detectable	
5	31.92	0.31
10	31.14	0.17
20	29.78	0.09
50	28.80	0.03
75	27.77	0.08
100	27.24	0.10

## 4 DISCUSSION

The aim of this study was to assess the possibility of using cells suspended in buffer‐based medium for molecular testing in a time window of 48 h after acquisition of cell sample. We have evaluated the quantity, quality, and the expression of two housekeeping genes at different storage points over a period of 48 h. Our results suggested that mutation detection on RNA level by qPCR is possible even though the cells were stored at ambient temperature for 48 h.

Using two cell lines as a cell model mimicking fresh unfixed cells was used to test our hypotheses. First, we determined the optimal extraction method of nucleic acids available in our laboratory. Among the tested extraction methods (extraction using Tri reagent, automated extraction, and manual extraction using spin columns), manual extraction using spin columns gave the most consistent results for both DNA and RNA in both cell lines and was thus selected for further experiments. Similarly, the superiority of the spin column extraction method over automated extraction method was also reported by Domínguez‐Vigil et al.^5^ Since clinical samples are often limited in quantity, we wanted to determine the minimal number of cells in a sample that still result in successful DNA and RNA extraction. We concluded that the smallest amount of cells for reliable fluorometric quantification of extracted DNA and RNA is 10^3^ cells. Despite the reliable measurements, the DNA and RNA quantities from this amount of cells were too low for reliable molecular testing. Extraction of 10^4^ cells yielded a relatively low amount of DNA and an adequate amount of RNA while extraction of 10^5^ cells resulted in high amounts of both DNA and RNA. In another study, it was also shown that 10^3^ cells were sufficient for successful analysis of short tandem repeats^33^ but––to our knowledge––no similar study included analysis of RNA expression.

Second, we tested the effect of storage conditions on the quality and quantity of nucleic acids on both cell lines separately. As seen in Figure 3, the quantity of extracted DNA depended mostly on the cell line, whereas the quantity of extracted RNA was somewhat depended on the cell line. Additionally, we saw some variability in the quantity of extracted nucleic acids due to storage temperature and duration; however, these changes were not consistent among all conditions. DNA and RNA biomass in a cell is influenced by growth conditions and varies even among cell lines of similar origin.^34^ RNA content in different cell lines of Chinese hamster ovary origin varied by 4%, while variation in DNA content was lower, approximately 1% of the total cell mass.^34^ Our results are consistent with these findings as variation in DNA quantity was lower than the variation in RNA quantity and since our cell lines were not of similar origin, greater variation in DNA and RNA content is expected compared to the variation between similar cell lines. Similarly, we can expect higher variability also in clinical samples as they are not of similar origin or are from different patients.

Surprisingly, regardless of storage conditions, the detection of housekeeping genes was possible in all samples in all tested time points. However, we observed the correlation between the gene expression levels and the storage duration (*ACTB*) and the storage temperature (*GAPDH*), thus confirming that the storage affects the quantification of gene expression levels. Stability of nucleic acids in cytological samples was according to our knowledge studied by one research group. Chong et al.^7^ did a study about RNA extraction methods for cervico‐vaginal cytology samples according to tube types, storage temperatures, and preservative solutions. However, Chong et al. did a comparison between samples stored at room temperature and freezer temperature, so our results cannot be compared, since the samples were in a preservative solution containing chemical for nucleic acid stabilization. On the other side Alves et al.^6^ did a study about finding the best RNA extraction technique, including minimization of time and cost to obtain samples of oral lesions utilized in qPCR, but unfortunately, they isolated the cells from smears and stored them at −80°C until extraction.

Lastly, we focused on determining the minimal percentage of mutated cells, required for successful determination of mutation. Establishing the minimum number of cells needed to allow a multigene massively parallel testing approach from cytology samples is crucial.^35^ There are several studies testing whether samples from fine‐needle aspiration are useful source for molecular testing. The adequacy of fresh samples is usually compared to smears or cell blocks, mostly on DNA level.^8^, ^36^, ^37^, ^38^ Using qPCR, we were able to detect the specific mutation in as low as 5% of mutated cells in a total amount of 10^5^ cells; moreover, the higher percentage of mutated cells resulted in earlier detection of the mutation. However, this threshold of mutated cells is dependent on the method used for the mutation detection––next‐generation sequencing (NGS) and qPCR are able to detect mutations at 1% allele frequency,^18^, ^35^, ^39^, ^40^ while mutation detection using Sanger sequencing is between 10% and 20%.^41^ Besides the increased sensitivity, NGS is suitable for analyzing several genes at once and for detecting gene fusions on RNA level.^42^, ^43^, ^44^ It requires less cytological material than previously used techniques; however, the price for NGS testing is often significantly higher compared to other methods (qPCR, FISH) as NGS testing requires special equipment and expensive reagents.

However, this study has some limitations that lessen wider usability of obtained results and demand additional experiments to confirm the results. Since the clinical samples are often limited in quantity and therefore very precious, we used samples from cell lines to conduct the experiments. We acknowledge that the appropriate extraction method and storage conditions for real clinical samples may differ from our results on cell line samples, since clinical samples contain cells from different cell types and may contain also other entities (cytokines, proteins, chemokines, …) that were not present in our samples. Moreover, determining the RNA integrity number would be beneficial information about the degradation of RNA confirming the obtained qPCR results. Another limitation is single‐gene approach for mutation detection that may be less precise than multi‐gene approach, although used approach is less financially demanding as multi‐gene approach requires either higher usage of reagents (several single‐gene tests) or more sophisticated equipment and/or reagents (NGS testing, qPCR with multiple probes).

Next steps in the research of this topic should include different cell lines as the variation in the quality and yield of nucleic acids was already observed among different harvests of one cell line and the variation between different cell lines is expected to be even higher. Moreover, the principles should be tested on diverse clinical samples as they are prone to greater variability due to biological factors and other molecular testing methods should also be included.

In conclusion, we showed that the manual extraction using spin columns is the most appropriate for fresh unfixed cell samples in cytology. Based on our results, 10^3^ cells in a sample is the minimal number of cells that gives measurable nucleic acid quantities, however; for adequate nucleic acid quantities, we recommend using at least 10^4^ cells for the extraction. As expected, the best result was obtained when the extraction of DNA and RNA was preformed immediately upon reception of the cell sample to the laboratory, however; reliable results can be achieved also after storing the samples for up to 48 h at room temperature. Moreover, quantity of extracted either DNA or RNA declined with higher temperatures and longer duration of storage but regardless of the storage conditions, we successfully detected both housekeeping genes and mutated gene using qPCR.

## CONFLICT OF INTEREST

The authors declare no conflict of interest.

## AUTHOR CONTRIBUTIONS

Conceptualization, M.S.F. and I.S.K.; methodology, A.D., G.G., and M.Ž.; validation, A.D., G.G., and M.Ž.; formal analysis, A.D., G.G., and M.Ž.; resources, M.S.F. and I.S.K.; writing—original draft preparation, A.D.; writing—review and editing, M.S.F. and I.S.K.; visualization, A.D. and M.Ž.; supervision, M.S.F. and I.S.K.; project administration, M.Ž; funding acquisition, M.S.F. and I.S.K. All authors have read and agreed to the published version of the manuscript.

## ETHICS STATEMENT

Not available.

## Precis

Using cell line model to mimic fresh cytology samples, we have determined the influence of storage conditions (time, temperature) on the quality and quantity of nucleic acids for cells suspended in a buffer‐based cell medium. Additionally, we assessed the minimal number of cells and minimal percentage of mutated cells per sample for successful molecular testing.

## Data Availability

Data available on request from the authors.
